# The Molecular Key to Understanding the Medical Ozone Action

**DOI:** 10.3390/ijms25116148

**Published:** 2024-06-03

**Authors:** Lamberto Re

**Affiliations:** 1 Clinical Pharmacology, Marche Polytechnic University, 60128 Ancona, Italy; lambertore@lambertore.com; 2 WFOT, Scientific Committee, 25128 Brescia, Italy

Currently, treatment with medical ozone (MO) is considered one of the most interesting and safe integrative options that can effectively complement many conventional medical therapies, mainly, but not exclusively, involving aging and pain [1].

Nevertheless, a clear mechanism of action in terms of pharmacological action is not well documented despite the numerous papers published in prestigious journals regarding its efficacy in many ailments [2,3,4,5].

This Special Issue aimed to gather experimental lines of evidence on the molecular aspects regarding extensive reported clinical data related to MO treatment, also considering findings at a neurological level [6,7].

This collection also sought to introduce some new experimental data exploiting the biological and medical action induced through adequate oxidative stimulation that, from a purely pharmacological point of view, is significantly different from the well-known Michaelis–Menten and mass-law equations defining the kinetics of drug–receptor interactions [8].

In our opinion, for MO to be recognized as an integrative medical approach, more clinical and basic studies should be performed to characterize the most intimate molecular mechanisms underlying its indirect action and its association with the reported clinical efficacy.

The involvement of biochemical pathways common to all cells of mammalian organisms on our planet is becoming an experimental model for a preventive as well as therapeutic approach in multiple diseases linked to oxidative stress and therefore to aging [9].

These discoveries can be of significant benefit both in terms of the increased life expectancy and considering the lack of resources to deal with many pathologies linked to the immune, cardiovascular, neurological, and other systems, mostly in the elderly population.

Recently, according to research coordinated by the Institute for Health Metrics and Evaluation and published by *The Lancet*, it has been reported that the global life expectancy has increased by 6.2 years overall [10], thus strengthening the need for new strategies aimed at maintaining a state of well-being and health for this segment of the population.

Experimental models linked to the activation of the Nrf2 metabolic pathway are more frequently established in the literature [11,12,13], mainly linked to oxidative stress.

Indeed, like other agents acting through pharmacological modulation [14], moderate oxidative stimulus comparable to the physiological “*eustress*” is also able to dissociate the Nrf2–Keap1 bond, thus activating a cascade of events responsible for several actions induced by the upregulation of thousands of genes [12,15].

The activation of Nrf2 promotes the initiation of numerous cellular responses capable of counteracting oxidative stress and many pathologies, including those in which chronic pain is the most common symptom [16].

Considerable evidence shows that NRF2 plays a central regulatory role, granting it the definition of “*pleiotropic transcription factor*” [17,18,19].

We hope that the success and quality of the studies included in this Special Issue will prompt similar initiatives to provide significantly more high-level scientific data to support this new therapeutic and preventive approach represented by MO, especially as an integrated and complementary treatment to conventional medicine.
